# The association between nurse managers’ work environment characteristics and psychological resilience: the mediating role of perceived VUCA environment

**DOI:** 10.1186/s12912-025-04062-0

**Published:** 2025-12-02

**Authors:** Duygu Gül, Betül Sönmez, Satı Birbudak, Aytolan Yıldırım

**Affiliations:** 1 Department of Nursing, Faculty of Health Sciences, Çanakkale Onsekiz Mart University, Çanakkale, Türkiye; 2 Department of Nursing Management, Florence Nightingale Faculty of Nursing, Istanbul University-Cerrahpaşa, University Neighborhood, Istanbul University-Cerrahpaşa Campus, Avcılar, Istanbul, 34320 Türkiye; 3 Civil Defence Unit, Dr. Siyami Ersek Thoracic and Cardiovascular Surgery Training and Research Hospital, Istanbul, Türkiye; 4 Department of Nursing, Faculty of Health Science, Istanbul Atlas University, Istanbul, Türkiye

**Keywords:** Nurse manager, Psychological resilience, Work environment, VUCA environment

## Abstract

**Background:**

Nurse managers often operate in environments volatile, uncertain, complex, and ambiguous (VUCA) healthcare environments. Psychological resilience is essential for sustaining effective nursing leadership and ensuring service quality under such conditions. This study examined the associations between nurse managers’ perceptions of work environment characteristics and psychological resilience, with a focus on the mediating role of perceived VUCA environment.

**Methods:**

This cross-sectional and correlational study was conducted with 388 nurse managers from seven regions of Türkiye were recruited through online snowball sampling. Data were collected using the Nurse Manager Information Form, the Nurse Manager Practice Environment Scale, the VUCA Scale, and the Brief Resilience Scale. In addition to Pearson’s correlation analysis, direct and indirect effects between variables were analyzed with Hayes PROCESS Macro (Model 4).

**Results:**

The direct effect of nurse managers’ overall work environment score on perceived VUCA environment was found to be significantly negative (β=-0.126, *p* = 0.025), while its effect on psychological resilience was significantly positive (β = 0.180, *p* < 0.001). Perceived VUCA environment had also a significant negative direct effect on psychological resilience (β=-0.370, *p* = 0.001). Finally, the indirect effect of the work environment on psychological resilience, partially mediated by the perceived VUCA environment, was statistically significant (β = 0.047, 95%CI [0.002, 0.094]). This finding indicates that a positive perception of the work environment among nurse managers increases their psychological resilience both directly and by reducing their perception of the VUCA environment.

**Conclusions:**

The findings of this study highlight the importance of supporting nurse managers in effectively navigating their work environments and enhancing their capacity to adapt to VUCA conditions, thereby enhancing psychological resilience. This study highlights the need for comprehensive strategies that take into account the challenges faced by nurse managers in VUCA environments and transform these challenges into opportunities for development in nursing management.

**Clinical trial number:**

Not applicable.

## Introduction

Nurse managers play a vital role in optimizing patient outcomes, enhancing nurse retention, delivering high-quality care, and advancing organizational performance. The efficacy of nurse managers is intrinsically linked to the cultivation of positive work environments that support their leadership functions [1]. The nurse manager work environment is conceptualized as the organizational context that supports practice environments and influences outcomes affecting nurses, patients, and healthcare institutions [2]. Distinct from the nurse work environment, the nurse manager work environment specifically emphasized the unique role and responsibilities of nurse managers. A supportive work environment for nurse managers can enhance organizational engagement and facilitate effective leadership, thereby creating conditions that better support frontline nursing staff [3]. A study conducted during the COVID-19 pandemic demonstrated that nurse managers who encouraged collaboration and aligned their teams around shared goals significantly increased nurses’ motivation to engage in learning and adapt to evolving clinical practices [4]. However, extant literature on nursing work environments has predominantly focused on frontline nurses, often neglecting the critical influence of nurse managers [5, 6].

The development of a supportive work environment is essential for the retention and sustained performance of nurse managers in leadership roles [7]. A recent study conducted in China found that, although nurse managers generally perceived their work environments positively, challenges remained concerning adequate budget resources, manageable workloads, and collegial nurse manager-physician relationships [8]. Empirical evidence demonstrated that positive work environments decreased burnout and turnover intentions [1, 5, 6, 9–11], as well as improved patient outcomes, including decreased mortality rates, enhanced survival, and shortened hospital stays [10]. Positive work environments also contribute to greater psychological resilience among nurses [11, 12], higher patient satisfaction [9], and better perceptions of care quality [5, 6]. Further, a supportive work environment has been identified as a significant predictor of nurse managers’ job satisfaction, retention, and performance outcomes [7, 13]. Importantly, such environments may increase nurse managers’ capacity to adapt to and manage unexpected or volatile circumstances [12]. A well-structured work environment can mitigate the adverse effects of a perceived VUCA environment by reducing the intensity of experienced uncertainty [14, 15]. Prior to the COVID-19 pandemic, studies have shown that a healthy work environments and high resilience levels mitigated burnout risk among nurse leaders [12]. More recently, Hamza et al. [16] established a positive relationship between favorable work environments and psychological resilience in nurse.

The VUCA framework—characterized by Volatility, Uncertainty, Complexity, and Ambiguity—has been widely adopted to conceptualize the multifaceted challenges encountered in dynamic healthcare settings [15]. Volatility refers to rapid and unpredictable changes that disrupt stability and diminish the ability to anticipate future conditions. It is characterized by unbalanced fluctuations that undermine predictability and contribute to organizational instability [17]. Uncertainty denotes a limited capacity to navigate future events and an increased likelihood of unexpected outcomes, reflecting a lack of clarity about potential scenarios and complicating decision-making processes [18]. Complexity involves dynamic interactions within an environment marked by chaos, where numerous interrelated variables contribute to problems, many of which are difficult to interpret or analyze [18, 19]. Ambiguity signifies a lack of clarity regarding causal relationships, timing, and consequences of events, impeding responses to fundamental questions such as “when,” “how,” and “why” an event occurred. In highly ambiguous contexts, the absence of reliable information and the limited applicability of past experience hinder accurate interpretation and prediction [18].

Adapting to these VUCA conditions is increasingly recognized as one of the most pressing challenges for healthcare systems worldwide [20, 21]. The COVID-19 pandemic presented a paradigmatic example of a VUCA environment, including rapid shifts in care delivery models, reallocation of nursing staff, reconfiguration of clinical units, and an increased patient volume—all of which intensified organizational uncertainty and operational complexity [22]. Within such contexts, nurse managers have been instrumental in the sustainability and quality of nursing services. During the COVID-19 pandemic, nurse managers reported key experiences such as adapting to rapid change, participating in organizational decision-making, managing uncertainty, prioritizing the bio-psycho-social well-being of nursing staff, ensuring continuity of care, and promoting teamwork and interprofessional collaboration [23]. In Jordan, despite the highly challenging and stressful conditions imposed by the pandemic, nurse managers indicated that exposure to novel and complex situations contributed to the development of their managerial competencies, particularly in areas such as decision-making, problem-solving, and critical thinking [24]. Their capacity for resilience is particularly important, given their leadership role in navigating ongoing transformations in hospital systems [25]. As healthcare delivery systems grow increasingly complex, the ability of nurse managers to effectively respond to these evolving challenges will be a decisive factor in the success and adaptability of healthcare institutions [26]. Empirical findings from the pandemic period indicate that nurse managers demonstrated considerable adaptability in the face of uncertainty, heightened stress, and rapidly evolving clinical circumstances [27, 28]. In such unprecedented situations—where reliance on prior experience alone proves insufficient—organizations that have cultivated crisis management capabilities are more likely to maintain operational continuity and competitive advantage. This is particularly crucial in VUCA environments, which are inherently unpredictable in both scope and duration [29]. In this context, a healthcare institution’s strategic advantage may increasingly depend on its ability to effectively mobilize and support the expertise of nurse managers. While extensive research has explored the nursing work environment [5, 6, 30, 31], studies that explicitly examine this environment through the lens of the VUCA framework remain limited [32]. Although existing literature has focused on outcome variables such as burnout, work engagement, and turnover intention [5, 6, 15], there is a notable gap in understanding how VUCA conditions function as contextual factors that shape the nursing work environment.

Psychological resilience is commonly conceptualized as a personal resource or individual capacity that facilitates adaptation to stress and adversity while promoting effective recovery. In nursing, where professionals often operate with overstretched and resource-constrained healthcare systems, resilience is increasingly recognized as a vital attribute. Enhancing resilience among nurses is associated with improved coping mechanisms, greater job satisfaction, workforce retention, and support for overall employee well-being [33]. Resilient nurses tend to effectively use both internal and external resources to navigate occupational challenges, thereby mitigating the adverse effects of stress, improving sleep quality, and sustaining psychological and physical well-being—factors that are strongly associated with optimal job performance. However, diminished resilience can impair stress management, increasing the risk of burnout and turnover intention [11]. This concern is particularly important for nurse managers, who frequently operate in high-stress environments with competing demands and organizational complexity [8, 34]. In today’s dynamic healthcare landscape—characterized by volatility, uncertainty, complexity, and ambiguity (VUCA)—the responsibilities of nurse managers have become increasingly multifaceted, highlighting the need for psychological resilience as a foundational competency [35, 36]. A high-quality nursing work environment is characterized by professional autonomy, effective leadership, collaborative team dynamics, and adequate resource availability—all of which are critical in fostering psychological resilience among nursing staff [37]. Empirical evidence has consistently demonstrated that supportive work environments positively influence nurses’ psychological resilience [11, 38, 39]. The Health Services Workplace Environmental Resilience Model, developed by Cusack et al. [40], highlights the relationship between the nursing work environment and psychological resilience. Carpio et al. [35] reported that the resilience of nurse managers has a direct impact on employee well-being, patient care outcomes, and institutional functioning. Low resilience among nurse managers is linked to reduced support for nursing staff, increased staff turnover, poorer clinical outcomes, and deteriorated work environments [36]. Nurse managers with higher resilience, on the other hand, are better equipped to manage complex situations, formulate adaptive coping strategies, and sustain effective leadership practices, which enhance job performance and organizational success [41]. In addition, findings from a study conducted during the COVID-19 pandemic found that nurse managers generally exhibited high resilience level, which were positively associated with the empowering behaviors perceived by their subordinates [42]. These results indicate the urgency of enhancing resilience in response to the growing prevalence of VUCA conditions in healthcare, particularly in the context of global crises such as the COVID-19 pandemic. VUCA conditions intensify the complexity of clinical decision-making by amplifying uncertainty, ambiguity, and systemic stressors, thereby reducing nurses’ resilience and increasing the risk of burnout [32].

## Theoretical framework

The theoretical framework for this study is the Complex Adaptive Systems (CAS) theory. A complex adaptive system is defined as a dynamic and self-regulating network of interacting agents that continuously evolve in response to internal and external stimuli [43]. In healthcare, CAS are conceptualized as networks of autonomous components whose behaviors are not entirely predictable. These components are interdependent, such that the actions of one influence and reshape the dynamics of others. According to CAS theory, healthcare functions as a non-linear system in which changes within a single domain may lead to gradual, system-wide effects over time [44]. The nursing work environment exemplifies the properties of a CAS, as it involves dynamic feedback loops, multi-actor interactions, and constant adaptation to external environmental changes. This paradigm recognizes the healthcare system as inherently complex, where agents—including nurse managers—play an active role in adapting, self-organizing, and influencing system-level change. The CAS framework offers an innovative theoretical lens for addressing these conditions [45]. The CAS theory supports the idea that positive work environments are not only achievable but essential for sustaining adaptive capacity and resilience within healthcare organizations [46]. In this context, nurse managers are expected to be adaptable, resilient, and durable professionals capable of responding effectively to the VUCA environments in which they operate. Accordingly, CAS theory offers nurse managers a conceptual framework for understanding, navigating, and managing the complexities inherent in healthcare systems. From this perspective, CAS theory supports the development of resilience and adaptability among nurse managers, enabling them to respond more effectively to dynamic and unpredictable practice environments [45]. This study contributes to the nursing literature by integrating the CAS framework with the VUCA perspective, offering a novel lens through which to examine the organizational and psychological challenges faced by nurse managers.

## This study

While considerable research has examined nurses’ work environments, there remains a limited number of studies that address these environments from the perspective of nurse managers [e.g., 8]. To date, and to the best of our knowledge, no studies have empirically investigated the relationships among nurse manager work environment characteristics, perceptions of VUCA conditions, and psychological resilience. Therefore, this study aims to address this gap by examining the associations between nurse managers’ perceived work environment characteristics and their psychological resilience, as well as the mediating role of perceptions of the VUCA environment in the relationship between work environment characteristics and resilience. Psychological resilience, which can serve as both an antecedent and an outcome variable in organizational research, was conceptualized as the dependent variable in this study. Specifically, within the context of a perceived VUCA environment, the study examined the impact of nurse managers’ work environment characteristics on their psychological resilience.

Based on the theoretical framework and above-mentioned findings, the following hypotheses (H) were developed:

### H1

Perceived characteristics of the nurse managers work environment are significantly associated with (a) perceptions of the VUCA environment and (b) psychological resilience.

### H2

Nurse managers’ perceptions of the VUCA environment are significantly associated with psychological resilience.

### H3

Perceptions of the VUCA environment mediate the relationship between perceived work environment characteristics and psychological resilience among nurse managers.

The findings of this study have potential to inform evidence-based strategies aimed at optimizing work environments within VUCA healthcare settings, with the objective of strengthening psychological resilience among nurse managers. Enhancing the quality of nurse managers’ work environments—particularly through interventions that serve as protective buffers against the adverse effects of VUCA conditions—is essential for fostering resilience as a sustainable professional resource. Such improvements are not only likely to improve job satisfaction and psychological well-being among nurses but may also contribute to better patient care outcomes.

## Methods

### Research design, setting, and sample

This study employed a cross-sectional and correlational design and adhered to the Strengthening the Reporting of Observational Studies in Epidemiology (STROBE) guidelines [47] in the reporting of its findings. The target population consisted of lower, middle and upper nurse managers employed in hospitals across Türkiye. Given the lack of a comprehensive registry or centralized data on the total number of nurse managers in Türkiye, the study used a non-probability snowball sampling method to recruit participants. Using a snowball sampling method, this study aimed to recruit nurse managers working in diverse hospital settings—state, foundation, or private—across both secondary care facilities (e.g., state and private hospitals) and tertiary care institutions (e.g., training and research hospitals, university hospitals, and city hospitals). Participants were drawn from urban centers and districts across all seven geographic regions of Türkiye. Eligible participants were nurse managers who held their positions for a minimum of six months (who have completed the trial and orientation period) and who provided informed consent through an online survey platform (Google Forms). As the total population was not known, the sample size was calculated using the formula for sample size with unknown population. Based on a 95% confidence level and a significance level of *p* < 0.05, a minimum sample size of 384 nurse managers was determined. A final sample of 388 participants was obtained, exceeding the minimum requirement. All participants met the inclusion criteria and were recruited through online distribution using the snowball sampling method.

### Data collection

Data collection was conducted between January 2022 and January 2023. The instruments included the Nurse Manager Information Form, the Nurse Manager Practice Environment Scale (NMPES), the VUCA Scale, and the Brief Resilience Scale (BRS). Of the two instruments used to assess environmental factors, the NMPES primarily evaluates components of the professional nursing work environment, while the VUCA Scale captures perceptions of change dynamics and interaction patterns within both the broader organizational and immediate work contexts. To facilitate data collection, informed consent and the survey instruments were digitized and hosted via Google Form. The survey link was then distributed to nurse managers through various social media platforms. Invited nurse managers were also encouraged to share the link with their colleagues.

### Instruments

#### Nurse manager information form

The form included a total of 12 items designed to capture demographic and professional characteristics. Personal variables included age, gender, marital status, and educational level. Professional variables included geographical region of employment, hospital type, total number of beds, total nursing staff, managerial position, years of experience in the nursing profession and institution, and work schedule.

#### Nurse manager practice environment scale (NMPES)

The Nurse Manager Practice Environment Scale (NMPES), originally developed by Warshawsky et al. [2] and adapted into Turkish by Tosun and Yıldırım [48], consists of 44 items across eight subscales. These subscales include: culture of patient safety (15 items), culture of meaning (4 items), culture of generativity (6 items), adequate budgeted resources (4 items), fair and manageable workload (3 items), constructive nurse manager-director relationships (6 items), collegial nurse manager-physician relationships (3 items), and effective nurse manager-unit staff relationships (3 items). Responses are recorded on a 6-point Likert scale (1 = strongly disagree, 6 = strongly agree), with higher scores indicating more favorable perceptions of the nurse managers’ work environment. All items are positively phrased. The total Cronbach’s alpha coefficient for the original scale was 0.97, with subscale coefficients ranging from 0.72 to 0.97 [2]. For the Turkish adaptation, Cronbach’s alpha values ranged from 0.512 to 0.876 across subscales, and 0.961 for the total scale [48]. In the present study, Cronbach’s alpha coefficients for the subscales ranged from 0.606 to 0.903, with a total scale coefficient of 0.952. The culture of meaning, adequate budgeted resources, and fair and manageable workload subscales demonstrated coefficients below the conventional threshold of 0.70. However, for subscales measuring subjective perceptions and consisting of a small number of items (typically three to five), internal consistency coefficients between 0.60 and 0.70 are generally regarded as indicating the lower limit of acceptability [49] (Table 2).

#### VUCA scale

The VUCA Scale, developed by Yurdasever [50], measures managers’ perceptions of the volatile, uncertain, complex, and ambiguous conditions within their work environments. The instrument developed as 20 items across four subscales: complexity (5 items), volatility (5 items), ambiguity (5 items), and uncertainty (5 items). In the validity analysis, the four-factor, 16-item scale—comprising complexity (5 items), volatility (5 items), ambiguity (4 items), and uncertainty (2 items)—accounted for 54.39% of the total explained variance. Indeed, the validation study by Çiçek and Erkasap [51], which employed a four-factor, 20-item version of the original scale, reported a total explained variance of 79.31%. Items are rated on a 5-point Likert scale (1 = strongly agree, 5 = strongly disagree), with all items positively phrased. Higher scores correspond to greater perceived exposure to VUCA conditions, while lower scores indicate lower perceptions of these environmental challenges. Yurdasever [50] reported Cronbach’s alpha coefficients between 0.537 to 0.723 for the subscales and 0.75 for the total scale. Çiçek and Erkasap [51] reported Cronbach’s alpha coefficients ranging from 0.901 to 0.951 for the subscales and 0.935 for the total scale. In the present study, CFA was performed on the original 20-item, four-factor scale. Items 1, 16, and 20 were removed due to low factor loadings, and an additional item was excluded due to an excessively high beta value. The final model confirmed a four-factor structure comprising 17 items: complexity (4 items), volatility (5 items), ambiguity (5 items), and uncertainty (3 items). Model fit indices demonstrated acceptable and good fit: χ²/df = 2.186, GFI = 0.932, AGFI = 0.904, CFI = 0.957, NFI = 0.92, IFI = 0.957, TLI = 0.946, and RMSEA = 0.055 [49]. In the present study, Cronbach’s alpha coefficients for the subscales ranged from 0.635 to 0.888, while the total scale demonstrated high internal consistency, with an alpha coefficient of 0.909. Although the complexity subscale demonstrated a Cronbach’s alpha below 0.70, this value (0.60 ≤ α ≤ 0.70) falls within the range generally regarded as the lower limit of acceptable for subscales containing few items and measuring subjective constructs, consistent with the criterion noted above [49] (Table 2).

#### Brief resilicence scale (BRS)

The Brief Resilience Scale, developed by Smith et al. [52] to measure individuals’ psychological resilience, was adapted into Turkish by Doğan [53]. The scale consists of six items within a single subscale and is rated on a 5-point Likert scale (1 = not suitable at all, 5 = completely suitable). Items 2, 4, and 6 are reverse-coded. After reversing the relevant items, higher scores indicate greater psychological resilience [53]. Cronbach’s alpha coefficients for the original scale ranged from 0.80 to 0.91 across four different samples [52]. In the Turkish adaptation study, the Cronbach’s alpha coefficient of the scale was reported as 0.83 [53]. In the present study, the Cronbach’s alpha coefficient for the total scale was 0.719.

### Data analysis

Data analysis was conducted using IBM SPSS Statistics 27 (IBM SPSS, Türkiye). Descriptive statistics, including mean, standard deviation, median, frequency, percentage, minimum, and maximum values, were used to summarize the data. There were not missing data. The normality of the data was assessed using skewness and kurtosis values. According to Hopkins and Weeks [54], a skewness and kurtosis value between + 3 and − 3 is considered indicative of a normal distribution. Based on this criterion, parametric tests were employed, assuming that the scale scores followed a normal distribution. Pearson correlation analysis and a mediation model were employed to examine the relationships between variables. Process regression analysis was used to assess the direct and indirect effects among the variables. The direct and indirect (mediation) effects were analyzed using Hayes’ PROCESS Macro Model 4 [55]. The mediation effect was assessed using the Bootstrap method with 2.000 samples at a 95% confidence interval. The Cronbach’s alpha coefficient was calculated to assess the reliability of the scale, and statistical significance was determined at the *p* < 0.05 level within the 95% confidence interval.

### Ethical considerations

Ethical approval for the study was obtained from the Istanbul University-Cerrahpaşa Social Sciences and Humanities Research Ethics Committee (Date: 05.10.2021; Issue No: 2021/231). In addition, permission to use the adapted versions of the scales was obtained via email from the respective authors. The study was conducted in accordance with the principles outlined in the Declaration of Helsinki. Data collection commenced following the approval of the ethics committee. Nurse managers were informed about the study through an Informed Consent Form integrated into the Google Form. The form included details on the study’s purpose, duration, voluntary participation, confidentiality, researcher contact information, and the right to withdraw at any time. Participants provided their informed consent by selecting the “I consent to participate in the research” option before proceeding to complete the data collection instruments.

## Results

### Descriptive characteristics of the participants

Nurse managers from all seven geographical regions of Türkiye participated in the study, with the highest representation from the Marmara region (62.9%, *n* = 244). The majority of participants were female (90.2%, *n* = 350). The mean age of the participants was 45.04 years (SD = 7.19), with an age range of 29 to 60 years and a median age of 50. The majority of participants were married (74.7%), and 59.0% held a bachelor’s degree (*n* = 229), while 31.2% had completed a master’s degree (*n* = 121). Among those with graduate education, the most common fields of specialization were health management (32.3%, *n* = 41) and nursing management (20.5%, *n* = 26), which together accounted for 52.8% of the reported graduate education areas (Table 1).

**Table 1:** Demographic and occupational characteristics of the participants (*N* = 388)

	Mean (SD)	Median (Min-Max)		*n*	%
**Age**	45.04 (7.19)	50 (29–50)	**Type of Hospital**		
			Training and Research Hospital	142	36.6
**Gender**	**n**	**%**	State Hospital	78	20.1
Female	350	90.2	State University Hospital	128	33.0
Male	38	9.8	Foundation/Private University Hospital	4	1.0
**Marital Status**			Private Hospital	19	4.9
Married	290	74.7	City Hospital	13	3.4
Single	98	25.3	Other	4	1.0
**Educational Status in Nursing**			**Total Number of Hospital Beds**		
Vocational High School of Health	11	2.8	≤ 250 beds	68	17.5
Associate Degree	21	5.4	251–500 beds	84	21.6
Bachelor’s Degree	229	59	501–750 beds	22	5.7
Master’s Degree	121	31.2	751–1000 beds	132	34.0
Doctorate Degree	6	1.5	1001–1250 beds	25	6.4
**Field of Master’s and Doctorate Education (** ***n*** ** = 127)**			1251–1500 beds	8	2.1
Surgical Nursing	5	3.9	1501–1750 beds	16	4.1
Pediatric Nursing	4	3.1	1751–2000 beds	6	1.5
Public Health Nursing	4	3.1	2001 and above beds	27	7.0
Nursing	2	1.6	**Total Number of Nurses in the Hospital (** ***n*** ** = 367)**		
Fundamentals of Nursing	4	3.1	≤ 250 nurses	84	22.9
Nursing Management	26	20.5	251–750 nurses	90	24.5
Internal Medicine Nursing	16	12.6	751–1250 nurses	158	43.1
Obstetrics and Gynecology Nursing	1	0.8	1251–1750 nurses	13	3.5
Mental Health Nursing	7	5.5	1751–2250 nurses	6	1.6
Health Management	41	32.3	2251 and above nurses	16	4.4
Other	13	10.2	**Position**		
Not Indicated	4	3.1	Director of Nursing Services	18	4.6
**Years of Professional Experience**			Director of Healthcare Services	16	4.1
≤ 10 years	70	18.0	Vice Director of Nursing Services	12	3.1
11–20 years	136	35.1	Vice Director of Health Services	7	1.8
21–30 years	154	39.7	Nurse Supervisor	23	5.9
31–40 years	28	7.2	Unit Charge Nurse	272	70.1
**The Region Where They Work**			Nurse Coordinator	29	7.5
Marmara	244	62.9	Other	11	2.8
Aegean	9	2.3	**Duration of Employment in the Hospital**		
Mediterranean	30	7.7	≤ 10 years	183	47.2
Black Sea	21	5.4	11–20 years	117	30.2
Eastern Anatolia	10	2.6	21–30 years	72	18.6
Southeastern Anatolia	13	3.4	31–40 years	16	4.1
Central Anatolia	61	15.7	**Work Schedule**		
			Fixed Day Shifts	335	86.3
			Fixed Night Shifts	3	0.8
			Rotating Shifts (day and night shifts)	50	12.9

SD: Standard Deviation, Min: Minimum, Max: Maximum

Among the participating nurse managers, 36.6% were employed at training and research hospitals (*n* = 142), 33.0% at state university hospitals (*n* = 128), and 20.1% at state hospitals (*n* = 78). Regarding institutional size, 34.0% worked in hospitals with a bed capacity of 751–1000 (*n* = 132), 21.6% in hospitals with 251–500 beds (*n* = 84), and 17.5% in hospitals with 250 or fewer beds (*n* = 68). In addition, 43.1% of nurse managers worked in hospitals with 751–1250 nurses (*n* = 158), 24.5% in hospitals with 251–750 nurses (*n* = 90), and 22.9% in hospitals with 250 or fewer nurses (*n* = 84). In terms of managerial roles, the majority served as unit charge nurse (70.1%, *n* = 272), followed by nurse coordinators (7.5%, *n* = 29) and nurse supervisors (5.9%, *n* = 23). The majority of nurse managers (39.7%, *n* = 154) had 21–30 years of professional experience, while 47.2% (*n* = 182) had been employed at their current institution for 10 years or less. In terms of work schedule, 86.3% (*n* = 335) reported working fixed day shifts, 12.9% (*n* = 50) worked rotating shifts, and 0.8% (*n* = 3) worked fixed night shifts (Table 1).

### Descriptive statistics and correlation matrix of the scales

Among the subscales of the NMPES, the lowest mean score was observed in adequate budgeted resources (M = 3.97, SD = 1.11), while the highest mean score was found in effective nurse manager–unit staff relationships (M = 5.28, SD = 0.72). The overall mean score for the total scale was 4.73 (SD = 0.70). In the subscales of the VUCA scale, the lowest mean score was observed in uncertainty (M = 2.86, SD = 1.05), while the highest mean score was found in volatility (M = 3.44, SD = 0.86). The overall mean score for the total scale was 3.16 (SD = 0.77). The mean score of the total BRS Scale was 3.44 (SD = 0.73) (Table 2).

**Table 2:** Descriptive statistics and correlation matrix of the scales

	1	2	3	4	5	6	7	8	9	10	11	12		13		14		15		
1. NMPES Culture of Patient Safety	(0.903)																			
2. NMPES Culture of Meaning	0.674**	(0.680)																		
3. NMPES Culture of Generativity	0.621**	0.763**	(0.772)																	
4. NMPES Adequate Budgeted Resources	0.569**	0.459**	0.423**	(0.677)																
5. NMPES Fair and Manageable Workload	0.617**	0.549**	0.528**	0.590**	(0.606)															
6. NMPES Nurse Manager-Director Relationships	0.852**	0.604**	0.537**	0.470**	0.535**	(0.882)														
7. NMPES Collegial Nurse Manager-Physician Relationships	0.557**	0.579**	0.618**	0.350**	0.409**	0.524**	(0.729)													
8. NMPES Nurse Manager-Unit Staff Relationships	0.476**	0.556**	0.595**	0.077	0.260**	0.379**	0.576**	(0.734)												
9. NMPES Total	0.942**	0.807**	0.782**	0.662**	0.717**	0.861**	0.690**	0.563**	(0.952)											
10. VUCA Complexity	-0.098	-0.111*	-0.133**	-0.004	-0.053	-0.145**	-0.085	-0.064	-0.115*	(0.635)										
11. VUCA Volatility	-0.085	0.025	0.024	0.038	0.020	-0.140**	-0.056	-0.029	-0.054	0.537**	(0.805)									
12. VUCA Ambiguity	-0.159**	-0.159**	-0.103*	0.087	-0.003	-0.203**	-0.149**	-0.193**	-0.147**	0.585**	0.615**		(0.888)							
13. VUCA Uncertainty	-0.028	-0.095	-0.070	0.182**	0.097	-0.090	-0.075	-0.256**	-0.037	0.435**	0.499**		0.656**		(0.756)					
14. VUCA Total	-0.122*	-0.105*	-0.083	0.090	0.015	-0.184**	-0.117*	-0.163**	-0.114*	0.759**	0.824**		0.901**		0.771**		(0.909)			
15. BRS Total	0.164**	0.275**	0.285**	-0.072	0.052	0.186**	0.298**	0.358**	0.217**	-0.324**	-0.244**		-0.381**		-0.419**		-0.412**		(0.719)	
Mean	4.71	4.84	4.88	3.97	4.59	4.74	4.93	5.28	4.73	3.26	3.44		3.01		2.86		3.16		3.44	
SD	0.80	0.80	0.75	1.11	0.99	0.98	0.91	0.72	0.70	0.81	0.86		1.05		1.05		0.77		0.73	
Min-Max	2–6	2–6	2–6	1–6	1–6	1–6	1–6	2–6	2–6	1–5	1–5		1–5		1–5		1–5		1–5	

Pearson Correlation, **p < 0.001, *p < 0.05, Cronbach’s alpha coefficients are shown in parentheses, NMPES: The Nurse Manager Practice Environment Scale, VUCA: Volatile, Uncertain, Complex, and Ambiguous, BRS: The Brief Resilience Scale, SD: Standard Deviation, Min: Minimum, Max: Maximum

When the correlations between the scales were evaluated, a significant weak negative correlation (0.100 < *r* < 0.399) was found between the VUCA complexity subscale and several subscales of the NMPES scale: culture of meaning (*r* = -0.111), culture of generativity (*r* = -0.133), constructive nurse manager-director relationships (*r* = -0.145), as well as the total NMPES score (*r* = -0.115) (*p* < 0.05). No significant correlation was found between the other subscales and the mean score of the VUCA complexity subscale (*p* >0.05). A weak negative correlation was found between constructive nurse manager–director relationships (*r* = -0.140) and the VUCA volatility subscale (*p* < 0.05). No significant correlation was found between the other subscales and the VUCA volatility subscale (*p* >0.05) [56] (Table 2).

A weak negative correlation was found between the VUCA ambiguity subscale and all NMPES subscales except for fair and manageable workload and adequate budgeted resources (*p* < 0.05) A significant correlation was found between adequate budgeted resources and the VUCA uncertainty subscale at a weak positive level (*r* = 0.182, *p* < 0.001), and between effective nurse manager-unit staff relationships and VUCA uncertainty at a weak negative level (*r* = -0.256, *p* < 0.001). No significant correlation was found between the other subscales and the VUCA uncertainty subscale (*p* >0.05). A weak negative correlation was determined between most subscales of the NMPES—except for culture of meaning, adequate budgeted resources, and fair and manageable workload (*p* >0.05)—and the total VUCA scale score (*p* < 0.05). Similarly, a weak negative correlation was found between the total NMPES score and the total VUCA score (*r* = -0.114, *p* < 0.05) [56] (Table 2).

Among the subscales of the NMPES Scale, all except adequate budgeted resources and fair and manageable workload (*p* >0.05) showed a weak positive correlation with the mean BRS score (*p* < 0.001). A statistically significant, a weak positive correlation was found between the mean total NMPES score and the mean BRS score (*r* = 0.217, *p* < 0.001). A significant negative correlation was found between the VUCA subscales and the mean BRS score, ranging from weak to moderate strength (e.g., uncertainty subscale, *r* = -0.419, 0.400 < *r* < 0.699) (*p* < 0.001). A moderately significant negative correlation was found between the total VUCA score and the BRS mean score (*r* = -0.412, *p* < 0.001) [56] (Table 2).

### Direct and indirect relationships between variables

Consistent with the proposed hypotheses, the direct effect of the NMPES total score on the VUCA Scale total score was statistically significant and negative (β₀ = -0.126, *p* = 0.025), supporting H1a. The direct effect of the mean VUCA total score on the BRS total score was also negative and statistically significant (β_0_ = -0.370, *p* = 0.000), supporting H2. Further analysis revealed that the total effect of the NMPES on psychological resilience, as measured by the BRS, was positive and statistically significant (β_0_ = 0.227, 95% CI: 0.125–0.330, *p* < 0.001). The direct effect of the NMPES on the BRS was similarly positive and significant (β_0_ = 0.180, 95% CI: 0.086–0.275, *p* < 0.001), supporting H1b. In addition, the indirect effect of NMPES on BRS score mediated by the VUCA score was statistically significant (β_0_ = 0.047, 95% CI: 0.002–0.094). This finding provides empirical support for H3, indicating that the VUCA partially mediates the relationship between nurse managers’ work environment characteristics and their psychological resilience (Table 3).

**Table 3:** The mediating role of VUCA perception in the effect of nurse manager work environment on psychological resilience

Model	Effect	β_0_	SE	t	*p*	β_1_	Lower Bound	Upper Bound	*R* ^2^	F
1	NMPES$\:\to\:$VUCA	-0.126	0.056	-2.247	0.025*	-0.114	-0.237	-0.016	0.013	5.048*
	VUCA$\:\to\:$BRS	-0.370	0.043	-8.545	< 0.001**	-0.392	-0.455	-0.285	0.199	47.796**
	*NMPES* $\:\to\:$ *BRS*									
	Total effect	0.227	0.052	4.362	< 0.001**	0.217	0.125	0.330	0.047	19.027**
	Direct effect	0.180	0.048	3.750	< 0.001**	0.172	0.086	0.275	0.199	47.796**
	Indirect effect	0.047	0.023	-	-	-	0.002	0.094	-	-

Process Hayes (Model 4), *p < 0.05, **p < 0.01, Durbin-Watson: 0.648, Tolerance: 0.987, VIF: 1.013, β_0_=Unstandardized Beta, β_1_=Standardized Beta, SE = Standard error, NMPES: The Nurse Manager Practice Environment Scale, VUCA: Volatile, Uncertain, Complex, and Ambiguous, BRS: The Brief Resilience Scale

## Discussion

This study examined the associations among nurse managers’ perceptions of their work environment, their experiences within a VUCA environment, and psychological resilience. Findings revealed that more favorable perceptions of the work environment were associated with higher levels of psychological resilience among nurse managers. In addition, the VUCA environment partially mediated this relationship, exerting a statistically significant negative indirect effect.

The quality of the professional practice environment is a well-established determinant of both individual nurse well-being and broader organizational outcomes [9]. In accordance with previous research, the current findings indicate that a supportive, empowering, and autonomy-promoting work environment can significantly enhance the psychological resilience of nurse leaders [12, 57]. Such environments likely contribute to adaptive coping mechanisms that buffer the psychological strain imposed by volatile and uncertain healthcare settings [35, 58]. Participants in this study generally reported positive perceptions of their work environment, as reflected in high scores on the NMPES scale. Specifically, strong scores were noted in relational subscales, such as collaboration with staff and physicians, and in reflecting a culture of meaning. The lowest scores were observed in the factor of adequate budgeted resources, consistent with findings from a pre-pandemic study conducted in Türkiye [48] and in China [8]. Notably, nurse managers in Türkiye reported lower overall NMPES scores compared to those in China. The results are not surprising since nurse managers rated their working environments more positively than staff nurses [59].

Leadership in the contemporary VUCA environment departs significantly from traditional hierarchical models. Instead, it demands capabilities supporting both individual and organizational resilience, particularly in navigating complex and uncertain conditions. Effective leadership in such contexts involves promoting interprofessional collaboration, supporting autonomous decision-making, and cultivating a culture of continuous learning and adaptation [22, 36]. Findings from this study indicate that nurse managers perceive the VUCA environment at a moderate level. This perception is especially pertinent in the post-COVID-19 era, where the urgency for agile adaptation has heightened and healthcare systems have grown increasingly complex and unpredictable. Among the VUCA subscales, volatility and complexity emerged as the most prominent characteristics identified by participants, suggesting heightened exposure to rapid changes and multifaceted challenges within their organizational settings.

The capacity of nurse managers to manage uncertainty and adapt to emergent conditions plays a critical role in enhancing resilience at a system level [25, 60]. Nurse managers in Türkiye face substantial performance demands, compounded by increasing service expectations, a persistent shortage of nursing personnel [61], and requirements to improve clinical outcomes. Within the framework of CAS theory, healthcare organizations are conceptualized as dynamic and self-organizing structures. In this model, the ability of leadership to facilitate flexible responses and foster open, collaborative interactions is essential for system-level adaptability and resilience [45]. The psychological resilience of nurse managers in the present study was found to be above average. This outcome suggests that the participants possess well-developed coping mechanisms that enable them to function effectively in high-pressure and rapidly evolving environments. Psychological resilience has been consistently identified as a protective factor against occupational burnout in nurse leaders [62, 63], and it is positively associated with improved leadership effectiveness and organizational performance [42]. While the finding of above-average resilience is encouraging, it also indicates the necessity for continued investment in leadership development programs and organizational strategies.

The findings of this study highlight that the importance of supportive organizational practices in enabling nurse managers to sustain effective leadership in healthcare systems characterized by high levels of uncertainty, complexity, and continuous change. Correlation analyses demonstrated that more favorable perceptions of the work environment were significantly positively associated with psychological resilience and significantly negatively associated with perceptions of the VUCA environment. Similarly, the perception of a more intense VUCA environment was negatively associated with psychological resilience. These findings are consistent with recent research by Gündüz et al. [57] and Okonkwo et al. [32], both of which emphasize the psychological demands on nurse leaders. The present study makes a noteworthy contribution by highlighting the relationship between perceived work environment quality and psychological resilience. This finding suggests that nurse managers’ perceptions of their external environment significantly influence their capacity to remain psychologically resilient. Evidence from the literature further supports that leadership style, organizational justice, a supportive climate, and individual psychological resources—such as self-efficacy, hope, and a sense of meaning—are key determinants of nurse resilience [62, 63]. Therefore, strategic improvements to the work environments and investing in leadership development programs designed to address the challenges of VUCA context may enhance both the resilience and leadership efficacy of nurse managers.

The results of the mediation analysis revealed that the perception of the VUCA environment partially mediates the relationship between the perceived quality of the work environment and psychological resilience among nurse managers. Specifically, a positive work environment was found to enhance psychological resilience both directly and indirectly by mitigating the perception of VUCA. This result is in line with the systematic review by Okonkwo et al. [32], which emphasizes the disruptive effects of VUCA conditions on nurse leaders and the buffering function of a supportive work environment in this situation. From a theoretical perspective, these findings align with the principles of CAS theory, which conceptualizes organizations as dynamic, self-regulating systems that are continuously evolving in response to internal and external stimuli [60]. The psychological resilience of nurse managers reflects the adaptability of a complex system characterized by a supportive work environment and perceptions of VUCA. The study findings suggest that both individual-level resilience and organizational-level adaptability—characterized by a supportive work environment and reduced perception of VUCA—are essential for maintaining effective nursing leadership in complex health systems. Notarnicola et al. [45] similarly highlight the critical role of nurse leaders guiding agile, learning-oriented, and interconnected organizational structures within the CAS paradigm. Accordingly, the present study provides valuable insight into how nurse managers can enhance their leadership effectiveness within a VUCA environment.

### Implications

This study provides important implications at the policy, organizational, and individual levels to promoting positive outcomes among nurse managers and healthcare institutions. At the policy level, incorporating resilience-building strategies into workforce planning is essential for health systems to proactively prepare for and effectively manage future VUCA conditions. Policymakers should develop comprehensive and integrated strategies to establish resilient organizational cultures that can anticipate and respond to crises. However, the findings highlight a continuing need for initiatives that strengthen positive work environments for nurse managers operating in VUCA contexts and enhance their psychological resilience. Furthermore, policymakers should cultivate VUCA-related competencies to better equip nurse managers for unpredictable and complex healthcare settings and to facilitate the rapid and effective transformation of work environments in response to crises.

At the organizational level, administrators, leaders, and human resource professionals should adopt supportive policies that reinforce reciprocal relationships and organizational cohesion. The findings provide evidence to inform nursing management practice and offer guidance for designing adaptive work environments that promote resilience among nurses. Creating environments in which nurse managers can effectively manage workloads, engage in decision-making, access professional development opportunities, enhance intra-team communication, optimize resource use, and receive sustained support for their leadership roles may not only mitigate the adverse effects of VUCA conditions but also foster greater resilience and team performance. Furthermore, establishing such supportive environments can enhance managerial efficiency by reducing the risk of burnout. Although nurse managers demonstrated relatively high psychological resilience, the identification of areas for further development underscores the need for institutional mechanisms that sustain and strengthen resilience. Mentorship programs led by experienced nurse leaders may enhance managers’ capacity to navigate organizational uncertainty, while coaching interventions focused on personal development and leadership can further support both individual and professional resilience. These targeted strategies may also alleviate emotional exhaustion by reducing the sense of isolation often reported by nurse leaders.

Leadership plays a critical role in navigating VUCA environments, and the development of essential leadership competencies in these contexts requires dedicated education and training. Therefore, it is recommended that healthcare organizations expand institutional training programs for nurse managers, focusing on stress management, resilience development, adaptive leadership, and flexible decision-making within VUCA conditions. Furthermore, workshops, leadership simulations, and crisis management training aimed at enhancing leadership capabilities is recommended. Such programs are expected to increase nurse leaders’ resilience to uncertainty.

Traditional leadership approaches are often inadequate for addressing the complexities and rapid transformations characteristics of contemporary healthcare environments. Therefore, institutional support mechanisms should be strengthened to enable nurse managers to sustain psychological resilience, guided by the principles of CAS theory within healthcare organizations. In this context, establishing multidisciplinary support systems is essential to prevent isolation in decision-making processes. In addition, fostering a culture of solidarity and collaboration among leaders enhances psychological resilience by facilitating experience sharing and strengthening collective problem-solving capacity. Organizations should therefore establish an environment that supports resilient leadership and encourages the application of flexible leadership practices consistent with the CAS framework.

The findings also emphasize the importance of nurse managers’ active engagement in creating and maintaining adaptive work environments that can respond effectively to VUCA conditions, thereby strengthening their psychological resilience. Nurse managers should assume proactive responsibility for enhancing their psychological resources at the individual level. By improving their personal competencies, they can mitigate the adverse effects of VUCA conditions on their resilience and sustain their capacity for effective leadership.

These findings further highlight key directions for future research. Subsequent studies could conduct detailed analyses of the individual subscales of perceived VUCA conditions to identify specific dimensions influencing resilience. Comparative research across diverse healthcare systems and cultural settings would enrich the understanding of contextual variations in nurse managers’ perceptions of resilience and VUCA. In addition, the effectiveness of interventions designed to enhance psychological resilience among nurse managers could be evaluated through experimental or quasi-experimental designs. Longitudinal research exploring temporal changes in resilience and their associations with organizational outcomes—such as staff retention, job performance, and well-being—would also provide valuable evidence for the development of sustainable leadership and workforce strategies.

### Limitations and strengths

This study has several limitations and strengths that should be considered when interpreting the findings. Data were collected using a cross-sectional design through self-administered online questionnaires completed by nurse managers. While this method facilitated broad geographic reach and increased convenience for participants, it also introduced certain limitations. However, given the non-random nature of snowball sampling, there is an inherent risk of sample homogeneity, which may limit the generalizability of the findings. The reliance on self-reported measures raises the potential for social desirability bias, which may have resulted in inflated or overly favorable responses. Furthermore, both perceptions of the work environment and psychological resilience are inherently dynamic constructs that may fluctuate in response to temporal and contextual factors. As such, the cross-sectional nature of the data limits the ability to capture these potential variations over time and precludes any inference of causality among the studied variables. Longitudinal research designs would provide more valuable insights into the directionality and stability of the observes associations. Despite these limitations, the study has several notable strengths. One key strength is the geographic and institutional diversity of the sample, which was enhanced by recruiting nurse managers from seven distinct regions across the country, therefore increasing the sampling method limitation. In addition, the use of an online data collection strategy enabled participation from a broader range of healthcare settings and minimized logistical barriers. However, the ongoing COVID-19 pandemic presented challenges to reach nurse managers, even online; snowball sampling contributed to an extended timeline for data collection.

## Conclusions

This study examined the relationship between the work environment, perceived VUCA conditions, and psychological resilience among nurse managers, who play a critical role in navigating volatile, uncertain, complex, and ambiguous healthcare settings. The findings indicate that a positive work environment significantly contributes to mitigating the adverse effects of VUCA conditions and enhancing psychological resilience. Furthermore, the results indicate that the relationship between the work environment and psychological resilience is partially mediated by perceived VUCA conditions. Strengthening perceptions of a supportive and adaptive work environment may therefore guide the development of organizational strategies aimed at maintaining system sustainability and promoting the psychological resilience of nurse managers.

## Data Availability

All the data used in the study are available from the first and corresponding authors on request.

## Abbreviations

CAS: Complex Adaptive Systems
VUCA: Volatile, Uncertain, Complex, and Ambiguous
NMPES: Nurse Manager Practice Environment Scale
BRS: Brief Resilience Scale
